# Macular Ganglion Cell Imaging Study: Covariate Effects on the Spectral Domain Optical Coherence Tomography for Glaucoma Diagnosis

**DOI:** 10.1371/journal.pone.0160448

**Published:** 2016-08-04

**Authors:** Jae Hoon Jeong, Yun Jeong Choi, Ki Ho Park, Dong Myung Kim, Jin Wook Jeoung

**Affiliations:** 1 Departement of Ophthalmology, Konyang University Hospital, Daejeon, Korea; 2 Chung-Ang University Graduate School, Seoul, Korea; 3 Department of Ophthalmology, Seoul National University Hospital, Seoul, Korea; 4 Department of Ophthalmology, Seoul National University College of Medicine, Seoul, Korea; University of Florida, UNITED STATES

## Abstract

**Purpose:**

To evaluate the effect of multiple covariates on the diagnostic performance of the Cirrus high-definition optical coherence tomography (HD-OCT) for glaucoma detection.

**Methods:**

A prospective case-control study was performed and included 173 recently diagnosed glaucoma patients and 63 unaffected individuals from the Macular Ganglion Cell Imaging Study. Regression analysis of receiver operating characteristic were conducted to evaluate the influence of age, spherical equivalent, axial length, optic disc size, and visual field index on the macular ganglion cell-inner plexiform layer (GCIPL) and peripapillary retinal nerve fiber layer (RNFL) measurements.

**Results:**

Disease severity, as measured by visual field index, had a significant effect on the diagnostic performance of all Cirrus HD-OCT parameters. Age, axial length and optic disc size were significantly associated with diagnostic accuracy of average peripapillary RNFL thickness, whereas axial length had a significant effect on the diagnostic accuracy of average GCIPL thickness.

**Conclusions:**

Diagnostic performance of the Cirrus HD-OCT may be more accurate in the advanced stages of glaucoma than at earlier stages. A smaller optic disc size was significantly associated with improved the diagnostic ability of average RNFL thickness measurements; however, GCIPL thickness may be less affected by age and optic disc size.

## Introduction

Optical coherence tomography (OCT) is invaluable for assessing the structural changes of glaucoma because it can provide quantitative and noninvasive in vivo measurements of macula and optic nerve head parameters.[1–3] The recent introduction of spectral domain OCT (SD-OCT) and measurement of the macular ganglion cell-inner plexiform layer (GCIPL) thickness using a ganglion cell analysis (GCA) algorithm of the Cirrus OCT (Carl Zeiss Meditec, Dublin, CA, USA) has enabled effective detection of glaucoma; further, the diagnostic efficacy is comparable with that seen when measuring the peripapillary retinal nerve fiber layer (RNFL) thickness and optic nerve head parameters.[4–7]

In general, for the evaluation of the SD-OCT device performance, diagnostic accuracies such as sensitivity, specificity, and receiver operating characteristic (ROC) curves are measured, and in most studies, the accuracy have been reported without taking into account the potential effects of covariates on test results. Although a single ROC curve represents the average performance of the test for all included participants, subjects in previous studies have frequently exhibited varying degrees of disease severity or differing values of other covariates, such as age, axial length (AL), and optic disc size. By using ROC regression methodology, several studies have demonstrated the effect of disease severity and other covariates on the performance of SD-OCT instruments in the diagnosis of structural damage in glaucoma.[8, 9]

Peripapillary RNFL thickness varies significantly with degree of myopia, age, ethnicity, AL, and optic disc area.[10–12] Consequently, thickness measurements of particular retinal layers in the macula could offer glaucoma detection ability comparable or superior to that of peripapillary RNFL thickness in highly myopic patients.[6, 13–15] Macular thickness may theoretically reflect retinal ganglion cell (RGC) loss better, because more than 50% of all RGCs are concentrated and multilayered in this region, and RGC bodies are 10 to 20-fold the diameter of their axons.[16–18] In addition, since the macula lies along the globe’s optical axis, the macula maybe less affected by myopic globe elongation than the optic disc and peripapillary structures. However, little is known about the effects of various covariates on the diagnostic power of SD-OCT parameters.

The purpose of the present study was to evaluate the influence of demographic and ocular variables, including optic disc size and disease severity, on glaucoma detection ability based on Cirrus HD-OCT measurements of macular GCIPL and peripapillary RNFL thickness.

## Materials and Methods

This investigation is based on the *Macular Ganglion Cell Imaging Study*, an ongoing prospective study of glaucoma patients and healthy individuals at the Glaucoma Clinic of Seoul National University Hospital. The study followed the tenets of the Declaration of Helsinki, and was approved by the Institutional Review Board of the Seoul National University Hospital (IRB No. H-1404-039-570). Written informed consent was obtained from all subjects.

### Study Subjects

All subjects underwent a complete initial ophthalmic examination, including best-corrected visual acuity, intraocular pressure (IOP) by Goldmann applanation tonometer, noncycloplegic auto refraction, silt lamp examination of the anterior segment, gonioscopy, fundoscopy by using a 90D lens, AL, stereoscopic color disc photographs and red-free RNFL photographs, standard automated perimetry using the Swedish interactive thresholding algorithm (SITA) standard of central 30–2 perimetry (Humphrey field analyzer II, Carl Zeiss Meditec, Dublin, CA, USA), and Cirrus HD-OCT. Patients with the following conditions were excluded from this study: best-corrected visual acuity < 20/40, IOP exceeding 21 mmHg, cylinder correction < –3.0 D or > + 3.0D, closed angle on gonioscopy, poor-quality OCT images (signal strength < 6 or movement artifact) and histories of ocular surgery other than cataract extraction, or structural diseases that could affect visual filed (VF) sensitivity.

Patients with glaucoma were defined as those with a glaucomatous VF defect confirmed by 2 reliable VF examinations and by the presence of glaucomatous optic disc cupping irrespective of the level of IOP. Cirrus HD-OCT results were not used to classify patients. Glaucomatous optic disc cupping was defined as neuroretinal rim thinning, notching, excavation, or retinal nerve fiber layer (RNFL) defect with corresponding VF deficit. A glaucomatous VF defect was defined as the consistent presence of a cluster comprising ≥3 contiguous non-edge points with a probability of occurring in <5% of the normal population (*P* < 5%), and one of these points having a probability of occurring in <1% (*P* < 1%) on the pattern deviation plot, or a result of outside the normal limits in the glaucoma hemifield test. Test for VF defects should be repeated at least two consecutive times. A test of VF can be called reliable when the fixation loss is <20% and the false-positive is <33%. Only reliable tests were included in the analysis.

Unaffected individuals did not have any glaucomatous optic nerve head damage, any visible RNFL defect, and any VF defect on 2 reliable standard automated perimetry tests. Cases in which both eyes of a patient or an unaffected control subject were eligible for the study, only 1 eye was chosen at random for inclusion. Preperimetric glaucoma patients who had definite structural glaucomatous change in optic disc or RNFL without any VF defects were excluded in this study for clarity.

### OCT Measurements

Two scans using the Cirrus HD-OCT, including the macular and optic disc cube protocol were acquired on the same day. The macular GCIPL and peripapillary RNFL thicknesses, and optic nerve head parameters were measured automatically using embedded analysis algorithms. The GCA algorithm (S1 Fig) detects the outer boundary of the RNFL as well as the outer boundary of the inner plexiform layer. The difference between these two outer boundaries yields a combined thickness of the retinal ganglion cell layer and inner plexiform layer.[19, 20] The optic disc cube program (S2 Fig) automatically detects the circle and positions it around the optic disc.[21] After the anterior and posterior boundaries of the RNFL are detected, the system calculates the RNFL thickness at each point on the circle.

The following OCT parameters were analyzed: average and minimum GCIPL thickness for the GCA algorithm, the average thickness for the peripapillary RNFL analysis, the disc area for the ONH analysis. Sectoral parameters of GCIPL or RNFL thickness were not included in analysis because specific sectoral parameter could be a bias in patients with unrelated topographic structural damage, and the best parameters were minimum GCIPL and average peripapillary RNFL thickness in the non-highly myopic[6] and early stage glaucoma[7] group in our previous *Macular Ganglion Cell Imaging Study*.

For correction of the automated SD-OCT disc area measurements, Bennett’s formula to correct for the magnification of the eye’s optical system[22] applied in the current study, and this approach was applied by Leung et al.[23] and Moghimi et al.[24] for evaluation of the relationship between the measured OCT image and the actual size of the disc, and the final corrected OCT disc size will be:

$$\begin{array}{l} \text{Corrected Cirrus HD-OCT disc area }\left({\text{mm}}^{2}\right)=\left(3.382^{2}\right)\times \left(0.01306^{2}\right)\times {\left(\text{AL}-1.82\right)}^{2}\times \left(\text{measured Cirrus HD-OCT optic disc area}\right) \end{array}$$

### Statistical Analysis

Differences in demographic and clinical characteristics including OCT parameters between glaucoma group and normal control group were assessed using independent t-tests and Fisher’s exact test.

A ROC regression modeling technique was carried out according to the methods of Alonzo and Pepe and of Janes and associates and details of the modeling procedure have been described in several publications.[25, 26] In the present study, the ROC regression model was fitted to assess the influence of the disease-specific covariate visual field index (VFI) and the common covariates age, spherical equivalent (SE), AL, and corrected optic disc area (CODA) on the diagnostic performance of OCT parameters as evaluated by ROC curves. The following ROC regression model was then fitted:

$$\text{ROC}\;\left(q\right)=\;\Phi \;\left[\alpha_{1}+\alpha_{2}\Phi^{-1}\left(q\right)+\beta_{1}\text{VFI}+\beta_{2}\text{Age}+\beta_{3}\text{Spherical equivalent}+\beta_{4}\text{Axial length}+\beta_{5}\text{Corrected optic disc area}\right]$$

where the coefficients *α*_1_ and *α*_2_ are the intercept and slope of the ROC curve, respectively, and Φ represents the normal cumulative function. If the coefficient (β) for covariate is greater than 0, then the variable positively influences the discrimination between subjects with disease. A negative coefficient indicates an inverse relationship. The area under the ROC curve (AUROC) can be obtained after the estimation of the parameters that indicating the type of test (the average thickness for the peripapillary RNFL analysis, average and minimum GCIPL thickness for the GCA algorithm) by using maximum likelihood probit models.

The probability level for statistical significance was set at 5%. Data were recorded and analyzed using SPSS for Windows version 18.0 (SPSS Inc., Chicago, IL, USA) and Stata trial version 13.0 (Statacorp, College Station, TX, USA).

## Results

Two hundred forty-nine eyes from 249 subjects (182 eyes with glaucoma and 67 normal control eyes) were included in this study. Thirteen eyes (5.2%) were excluded from the study because of scans showing algorithm segmentation failure (*n* = 5), signal strength <6 (*n* = 4), and artifacts due to eye movements or blinking (*n* = 4). Therefore, 173 eyes from 173 patients newly diagnosed with glaucoma and 63 eyes of 63 unaffected individuals (control group) were included in the final study. Demographic and quantitative measurements of ocular characteristics from the glaucoma and unaffected control groups are presented in Table 1. These groups did not differ significantly with respect to demographic data, including mean age and gender ratio. In addition, ocular characteristics including visual acuity, IOP, and SE did not differ significantly between groups. However, AL was longer (*P* = .011) in the glaucoma group (24.69 mm) than in the unaffected control group (24.12 mm). There were significant differences in global indices (mean deviation, pattern standard deviation, and VFI) of standard automated perimetry and OCT parameters between groups, with the exceptions of optic disc area and CODA. The average VFI score of the 173 glaucomatous eyes was 89.0% (median, 93%; first quartile, 84.5%; third quartile, 97%).

**Table 1:** Demographic and ocular characteristics in eyes with glaucoma and unaffected control groups.

Characteristic	Glaucomatous eyes (n = 173)	Unaffected eyes (n = 63)	*P* value
Age, yrs	55.16 (11.28)	53.71 (11.73)	.389*
No. of females (%)	86 (49.7)	33 (52.4)	.769^†^
Visual acuity, logMAR	0.07 (0.12)	0.06 (0.11)	.561*
Intraocular pressure, mmHg	13.08 (2.45)	13.23 (2.19)	.682*
Spherical equivalent, diopters	–2.28 (3.14)	–1.78 (3.69)	.298*
Axial length, mm	24.69 (1.48)	24.12 (1.58)	.011*
Index of visual field			
Mean deviation, decibels	–4.19 (3.45)	–0.47 (1.53)	< .001*
Pattern standard deviation, decibels	6.74 (4.12)	1.85 (0.52)	< .001*
Visual field index, %	88.99 (9.85)	99.27 (1.08)	< .001*
Cirrus OCT parameters			
Optic disc area, mm^2^	1.95 (0.41)	2.07 (0.43)	.067*
Corrected Optic disc area^‡^, mm^2^	1.98 (0.41)	2.00 (0.47)	.730*
Average RNFL thickness, μm	76.95 (10.49)	93.05 (8.80)	< .001*
Average GCIPL thickness, μm	71.66 (7.58)	79.21 (11.68)	< .001*
Minimum GCIPL thickness, μm	60.45 (11.25)	76.81 (6.83)	< .001*

The data are given as mean (standard deviation)

LogMAR = logarithm of the minimum angle of resolution; RNFL = retinal nerve fiber layer; GCIPL = ganglion cell-inner plexiform layer

*Student *t*-test

^†^Fisher exact test

^‡^Meassured Cirrus SD-OCT optic disc area)×(3.382^2^)×(0.01306^2^)×(Axial length—1.82)^2^

The analysis of the ROC regression model investigating the effect of demographic and ocular factors on the accuracy of Cirrus HD-OCT parameters is presented in Table 2. For average peripapillary RNFL thickness, a better diagnostic performance was significantly associated with younger age, longer AL, and a small CODA, in addition to lower VFI. However, for GCIPL thickness parameters, with the exception of VFI, longer AL was significantly associated with a better diagnostic performance for average GCIPL thickness only.

**Table 2:** Results of the receiver operating characteristic regression for retinal nerve fiber layer and ganglion cell-inner plexiform layer parameters.

Factor	Coefficients	95% Confidence interval	*P* value
Average RNFL			
(Intercept)	3.3567	(–1.2295, 7.9429)	.151
Ф^-1^(t)	0.9892	(0.7874, 1.1909)	< .001
**Visual field index**	–0.0478	(–0.0644, –0.0313)	**< .001**
**Age**	0.0160	(–0.0311, –0.0009)	**.038**
Spherical equivalent	0.0089	(–0.0760, 0.0937)	.838
**Axial length**	0.1848	(0.0067, 0.3629)	**.042**
**Corrected optic disc area**	–0.4723	(–0.8566, –0.0880)	**.016**
Average GCIPL			
(Intercept)	–0.1257	(–4.7120, 4.4606)	.957
Φ^-1^(t)	1.7437	(1.3881, 2.0993)	< .001
**Visual field index**	–0.0451	(–0.0616, –0.0287)	**< .001**
Age	0.0122	(–0.0029, 0.0273)	.112
Spherical equivalent	0.0090	(–0.0759, 0.0938)	.836
**Axial length**	0.1866	(0.0085, 0.3647)	**.040**
Corrected optic disc area	0.0104	(–0.3706, 0.3915)	.957
Minimum GCIPL			
(Intercept)	8.0907	(3.4425, 12.7388)	.001
Φ^-1^(t)	0.7010	(0.5580, 0.8439)	< .001
**Visual field index**	–0.0578	(–0.0747, –0.0410)	**< .001**
Age	0.0118	(–0.0033, 0.0268)	.125
Spherical equivalent	–0.0178	(–0.1026, 0.0671)	.681
Axial length	–0.0783	(–0.2555, 0.0989)	.386
Corrected optic disc area	–0.0030	(–0.3840, 0.3781)	.988

RNFL = retinal nerve fiber layer; Φ = probit function; GCIPL = ganglion cell-inner plexiform layer

Boldface values in the table indicate those results which are statistically significant at the *P* < 0.05 level.

**Fig 1:**
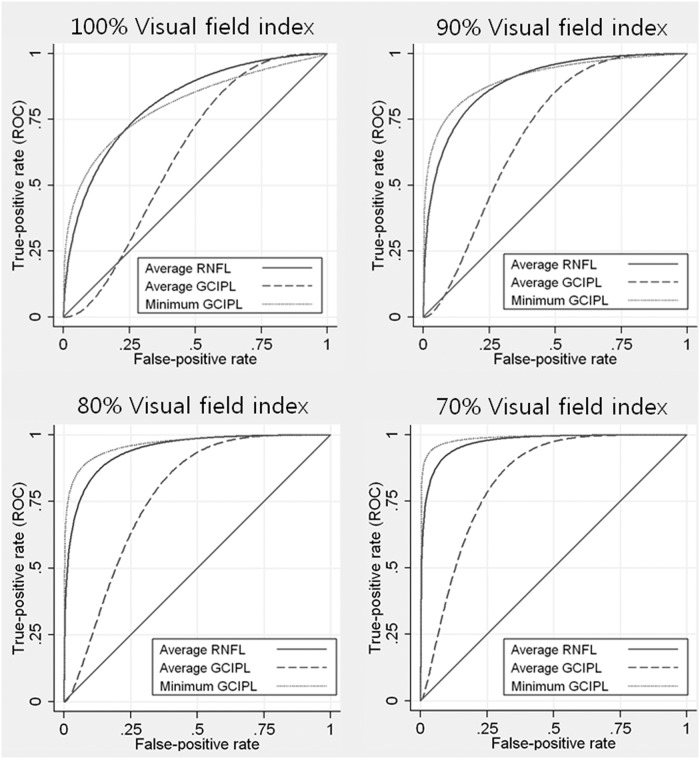
Receiver operating characteristic curves for the Cirrus HD-OCT parameters for arbitrary values of visual field index percentage, according to the regression model. Age, spherical equivalent, axial length, and corrected optic disc area were fixed at 55 years, –2.15 D, 24.5 mm, and 2 mm^2^, respectively. Receiver operating characteristic curves at a visual field index of 100% (Top left), 90% (Top right), 80% (Bottom left), and 70% (Bottom right) are shown. The solid, dotted, and broken lines indicate average RNFL, average GCIPL, and minimum GCIPL thickness, respectively.

The severity of disease, as measured by the VFI, had a significant influence on the diagnostic performance of all Cirrus HD-OCT parameters, as indicated by the statistically significant value attributed to the coefficient representing severity (*P* < .001). Fig 1 shows ROC curves for peripapillary RNFL and GCIPL thickness for arbitrary chosen levels of VFI percentage, and for an age of 55 years, an SE of –2.15 D, an AL of 24.5 mm, and a CODA of 2 mm^2^, as calculated from the regression model. Sensitivities were significantly higher for worse disease severities, the coefficients of VFI for average peripapillary RNFL, average GCIPL, and minimum GCIPL was –0.0478, –0.0451, and –0.0578, respectively (Table 2). The AUROC of average peripapillary RNFL for arbitrary VFIs of 100%, 90%, 80%, and 70% was 0.8158, 0.8926, 0.9431, and 0.9727, respectively, and similar effects were observed in the ROC curves of average and minimum GCIPL thickness (Table 3).

**Table 3:** AUC for OCT parameters and arbitrary values of disease severity, according to the regression models.

Visual field index (%)	Average RNFL	Average GCIPL	Minimum GCIPL
100	0.8158	0.6189	0.8103
90	0.8926	0.7009	0.9119
80	0.9431	0.7738	0.9661
70	0.9727	0.8354	0.9892

Data are AUCs for age of 55 years, spherical equivalent of -2.15 D, axial length of 24.5 mm, and corrected optic disc area of 2 mm^2^

RNFL = retinal nerve fiber layer; GCIPL = ganglion cell-inner plexiform layer

**Fig 2:**
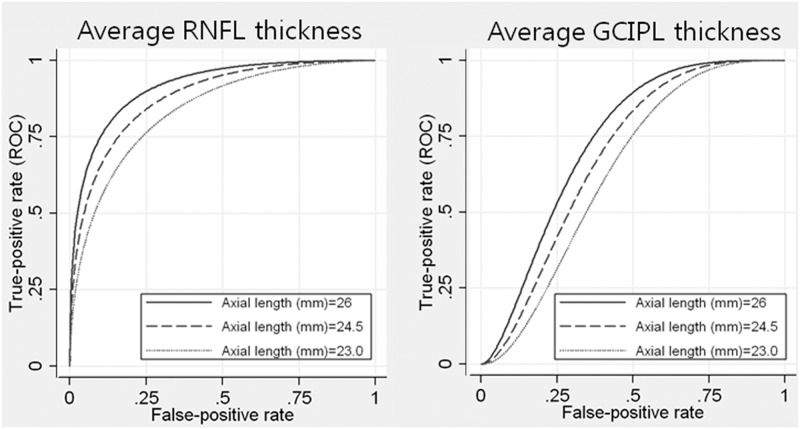
Receiver operating characteristic curves for the average RNFL (Left) and average GCIPL thickness (Right) parameter for arbitrary values of axial length, according to the regression model. Age, spherical equivalent, visual field index, and corrected optic disc area were fixed at 55 years, –2.15 D, 92%, and 2 mm^2^, respectively. The broken, dotted, and solid lines indicate axial lengths of 23.0 mm, 24.5 mm, and 26.0 mm, respectively.

AL was significantly associated with the diagnostic performance of average peripapillary RNFL and GCIPL thickness, but not with minimum GCIPL thickness. Both average peripapillary RNFL and GCIPL thickness performed better in longer eyes, as indicated by the positive and statistically significant coefficient associated with AL (0.1848 and 0.1866, respectively; *P* < .05); ROC curves for arbitrary AL values are shown in Fig 2. The AUROC of average peripapillary RNFL for arbitrary ALs of 23.0 mm, 24.5 mm, and 26.0 mm was 0.8351, 0.8795, and 0.9147, respectively. A similar effect was observed in the ROC curves of average GCIPL thickness; we obtained an AUROC of 0.6342, 0.6852, and 0.7328, for AL values of 23.0 mm, 24.5 mm, and 26.0 mm, respectively.

**Fig 3:**
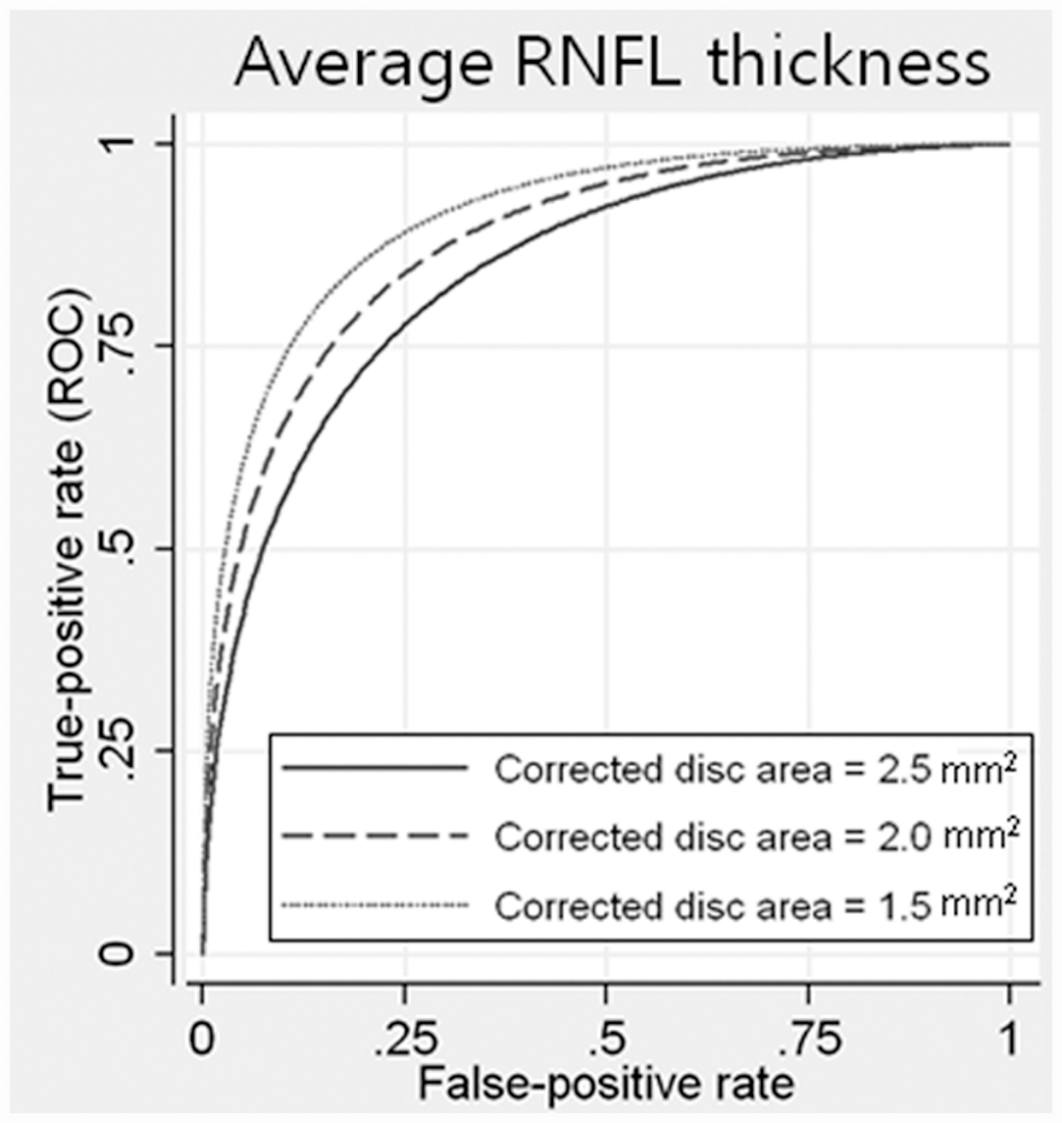
Receiver operating characteristic curves for the average RNFL thickness parameter for arbitrary values of corrected optic disc area, according to the regression model. Age, spherical equivalent, axial length, and visual field index were fixed at 55 years, -2.15 D, 24.5 mm, and 92%, respectively. The broken, dotted, and solid lines indicate 1.5 mm^2^, 2.0 mm^2^, 2.5 mm^2^ in corrected optic disc area, respectively.

CODA and age were significantly associated with diagnostic performance of average peripapillary RNFL thickness only, but not with GCIPL thickness. The diagnostic performance of average peripapillary RNFL thickness was significantly better for smaller optic discs (coefficient = –0.4723; *P* = .016) and younger ages (coefficient = –0.016; *P* = .038). ROC curves for arbitrary corrected optic disc area values are shown in Fig 3; the AUROC of average peripapillary RNFL for arbitrary CODA of 1.5 mm^2^, 2.0 mm^2^, and 2.5 mm^2^ was 0.9100, 0.8795, and 0.8423, respectively.

## Discussion

When evaluating the structural integrity of glaucoma, OCT provides valuable information on peripapillary RNFL thickness and optic nerve head parameters. In addition, parameters on the segmentation of individual retinal layers in the macular region have been recently utilized in the assessment of glaucoma. In this study, using ROC regression methodology, we investigated to the influence of multiple covariates including demographic and ocular characteristics on glaucoma detection ability based on Cirrus HD-OCT measurements.

Our findings relating to the effect of disease severity on SD-OCT measurements are consistent with previous studies using ROC regression methodology. Leite et al.[8] demonstrated that the performance of the different Cirrus RNFL thickness parameters tended to be more sensitive in advanced stages of glaucoma, and VFI was utilized as a descriptor of disease severity. Nakano et al.[9] reported that better mean deviation values were significantly associated with a worse diagnostic performance of macular ganglion cell complex and circumpapillary RNFL measurements on the RTVue-100 OCT (Optovue Inc., Fremont, CA, USA). In our study, VFI was the only factor that had a significant effect on all Cirrus HD-OCT parameters (average peripapillary RNFL, average GCIPL, and minimum GCIPL thickness), and the probability level was more significant than the other covariates (*P* < .001).

The AUROCs of average peripapillary RNFL and minimum GCIPL thickness were higher than that of average GCIPL, and the AUROC of minimum GCIPL was comparable to that of average RNFL thickness. Although the purpose of our study did not involve the comparison of the diagnostic accuracy between macular GCIPL and peripapillary RNFL, these results are similar to those of our previous studies.[6, 7] The minimum GCIPL thickness was designed to be sensitive to focal RGC loss, and does not depend on averaging the thickness of the area, this parameter can be completely free of the averaging effect.[5, 27] Since our glaucomatous participants were in the early stage of disease, the average mean deviation and VFI was –4.19 dB (range: –13.98–1.31) and 89% (range: 58–99%), respectively, the minimum GCIPL parameter showed better a glaucoma diagnostic performance than average GCIPL and was comparable to that of the average RNFL.

In the present study, a longer AL was associated with a better diagnostic accuracy with average RNFL and average GCIPL thickness; however, this contradicts the findings of a previous study using the ROC regression methodology. Nakano et al.[9] found that a longer AL was associated with a worse diagnostic performance for all circumpapillary RNFL and global loss volume in macular ganglion cell complex parameters. To explain this, the degree of myopia in participants requires consideration. The Nakano et al.[9] included highly myopic eyes (< –6 D) in nearly half (90/181) of their glaucomatous participants, with an average SE and AL of –9.5 D and 27.5 mm, respectively. However, in our study, the average SE and AL was –2.1 D and 24.5 mm, respectively, and the proportion of high myopic eyes was 15% (36/236).

Although previous reports regarding the relationship between average peripapillary RNFL measurements and myopia are controversial, peripapillary RNFL thickness typically tends to be thinner in highly myopic patients than in the unaffected population,[10–12] and highly myopic eyes have shown significantly different diagnostic accuracies compared with emmetropic eyes.[15] In the present study, AL showed a negative correlation with the peripapillary RNFL thickness in both the glaucoma (Pearson correlation coefficient = –0.289) and unaffected control groups (Pearson correlation coefficient = –0.407). Consequently, our results suggest that AL may have an effect on both peripapillary RNFL and macular GCIPL parameters; further research is necessary to establish the correlation between AL and glaucoma diagnostic performance.

A smaller optic disc size was significantly associated with a better diagnostic performance with average RNFL thickness, and it should be noted that we used CODA to minimize the magnification effects. Our result may be similar with previous studies in that the size of optic disc may have influenced the disc margin definition algorithms. The optic disc cube protocol of the SD-OCT device uses a 3.46 mm fixed-diameter circle; the optic disc size affects peripapillary RNFL thickness as it decreases with increasing distance from the disc margin.[12, 28–31] In contrast, the diagnostic performances of GCIPL parameters were not significantly affected by optic disc area. The present results indicate that peripapillary RNFL thickness may be unreliable, and that the combination of GCIPL parameters could improve glaucoma detection in eyes with large optic disc sizes.

Age was a significant predictor for diagnostic performance with average RNFL thickness, but not with GCIPL parameters. A potential explanation for this is the relationship between RNFL and age; however, previous studies have reported contradictory results. Most OCTs studies have reported that the RNFL thickness becomes thinner with increasing age;[10, 32–34] however, Rao et al.[35] reported that age did not influence RNFL measurements. In histological studies, although Balazsi et al.[36] reported a negative correlation between age and axonal number, in later work Mikelberg et al.[37] and Repka and Quigley[38] found no correlation. In addition, significant effects of age on macular thickness measurements have been reported.[35, 39, 40] To summarize, we propose that diagnostic accuracies of GCIPL thickness are less vulnerable to variables such as optic disc size and age, compared with that of peripapillary RNFL.

In general, SE is negatively correlated with AL. However, SE had no effect on the diagnostic performance of any of the Cirrus HD-OCT parameters in the present study, whereas AL significantly influenced average RNFL and GCIPL thickness. This was potentially due to the fact that two thirds of eyes included in the study had a SE < –3.0 D or > +3.0 D (data not shown), and we may have failed to detect a significant effect of SE on the ability of the Cirrus HD-OCT to detect glaucoma due to the narrow range of variables.

This study has some limitations that need to be considered. First, signal strength could have biased our results. Rao et al.[35] reported that signal strength significantly influenced all optic nerve head measurements by SD-OCT and suggested that signal strength may be potential a confounding factor in the association between age and RNFL measurements. Our study used a signal strength of at least 6 for inclusion; however, we cannot consider the association between signal strength and OCT measurements, or between signal strength and age. Second, in contrast to optic disc size, we did not revise the effect of angle between the scanning beam and the macula. Hariri et al.[41] demonstrated that macular thickness and volume measurements obtained by SD-OCT may be affected significantly by the position of the scanning beam and the angle of incidence on the retina. In fact, the tilting scan effect with an angle of greater than 15 degrees was observed in four eyes (three eyes with glaucoma and one control eye) with fundus features of high myopia or staphylomatous change, however, they comprised < 2% of the total subjects. Third, there was a small but significant difference in AL between the glaucomatous and unaffected control groups. Therefore, a control for the signal strength and the intergroup difference in AL may provide a clearer interpretation of the effect of AL and age to the diagnostic accuracy of SD-OCT. Another limitation of this study is the lack of generalizability of the findings since they were obtained from a highly population-specific (Korean) subjects.

In our investigation for significant factors that affect the diagnostic performance of OCT parameters, we demonstrated that disease severity has a significant effect not only on the diagnostic performance of average peripapillary RNFL, but on that of average and minimum GCIPL thickness. The ability of average peripapillary RNFL thickness to detect glaucoma was significantly affected by optic disc size. The diagnostic performance of average and minimum GCIPL thickness may be less affected by age and optic disc size than by peripapillary RNFL. Furthermore, AL was not significantly associated with the ability of minimum GCIPL thickness to detect glaucoma. Our findings may have significant implications for the use of SD-OCT in glaucoma diagnosis.

## Supporting Information

S1 Fig Ganglion cell analysis of Cirrus HD-OCT. Detection and measurement of the macular GCIPL thickness within a 6 × 6 × 2 mm^3^ cube centered on the fovea. Two ellipses were shown in GCIPL thickness map (top). The elliptical annulus has an inner vertical diameter of 1 mm and an outer diameter of 4 mm, and an inner horizontal diameter of 1.2 mm and an outer diameter of 4.8 mm. The deviation map (bottom left) and significance map (bottom right) indicate that the GCIPL thickness is within the normal limits.
(TIF)

S2 Fig Optic nerve head and RNFL analysis of Cirrus HD-OCT. RNFL thickness map (top left) obtains optic disc images through a 6 × 6 × 2 mm^3^ cube of data using 200 × 200 axial scans. A 3.46-mm diameter circle around the optic disc is shown on the RNFL deviation map (bottom left). The Cirrus HD-OCT system and the algorithm automatically detect the circle and extract a B-scan from it. Significance map (Right) of the same eye shown in S1 Fig. indicates that the RNFL thickness is within the normal limits.
(TIF)
