# Supplementary figures and images for: Macular Ganglion Cell Imaging Study: Covariate Effects on the Spectral Domain Optical Coherence Tomography for Glaucoma Diagnosis
Source: PLoS One. 2016 Aug 4;11(8):e0160448. doi: 10.1371/journal.pone.0160448 (PMC4974000; doi:10.1371/journal.pone.0160448)

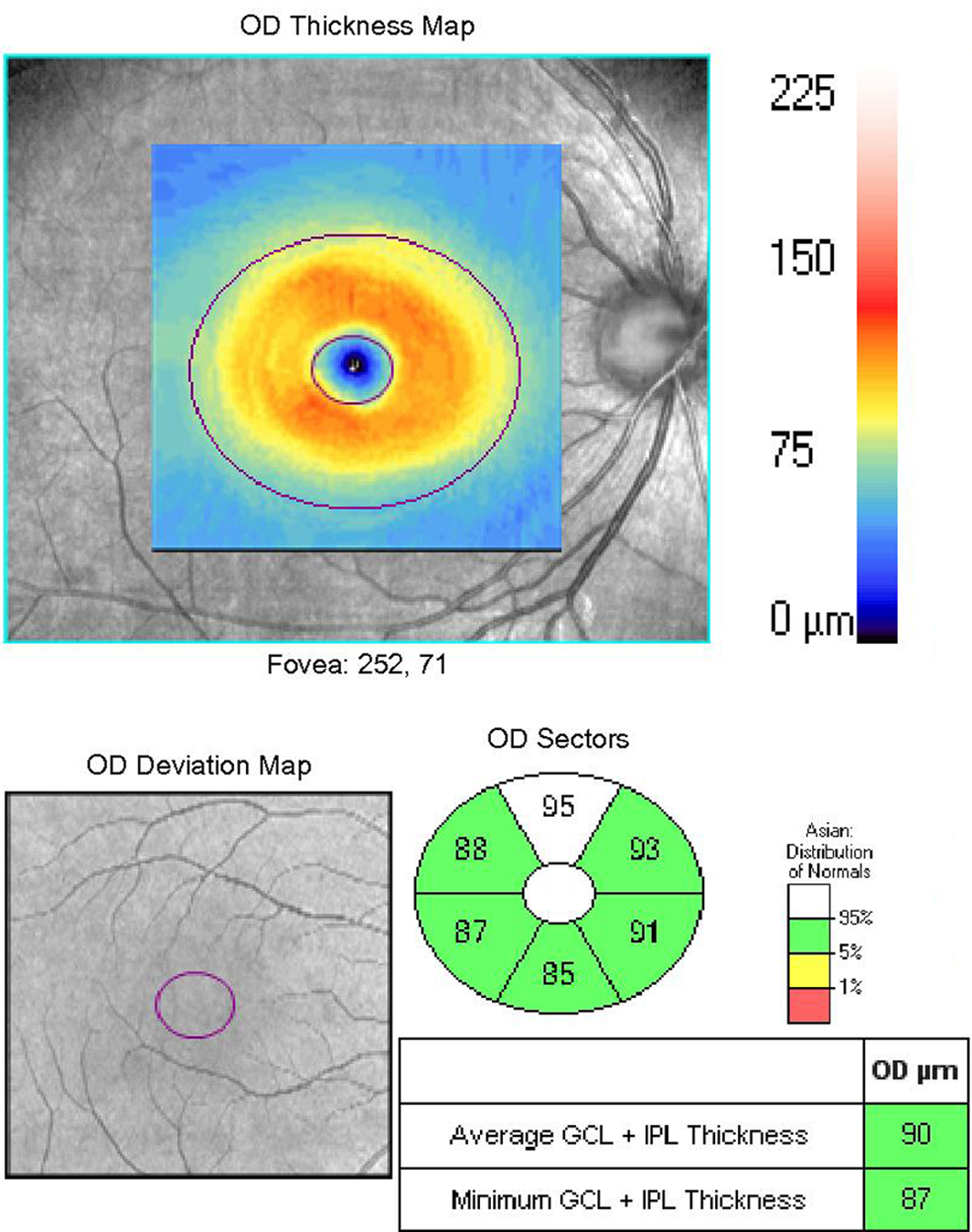

Supplement: S1 Fig — Detection and measurement of the macular GCIPL thickness within a 6 × 6 × 2 mm3 cube centered on the fovea. Two ellipses were shown in GCIPL thickness map (top). The elliptical annulus has an inner vertical diameter of 1 mm and an outer diameter of 4 mm, and an inner horizontal diameter of 1.2 mm and an outer diameter of 4.8 mm. The deviation map (bottom left) and significance map (bottom right) indicate that the GCIPL thickness is within the normal limits. (TIF) [file pone.0160448.s001.tif]

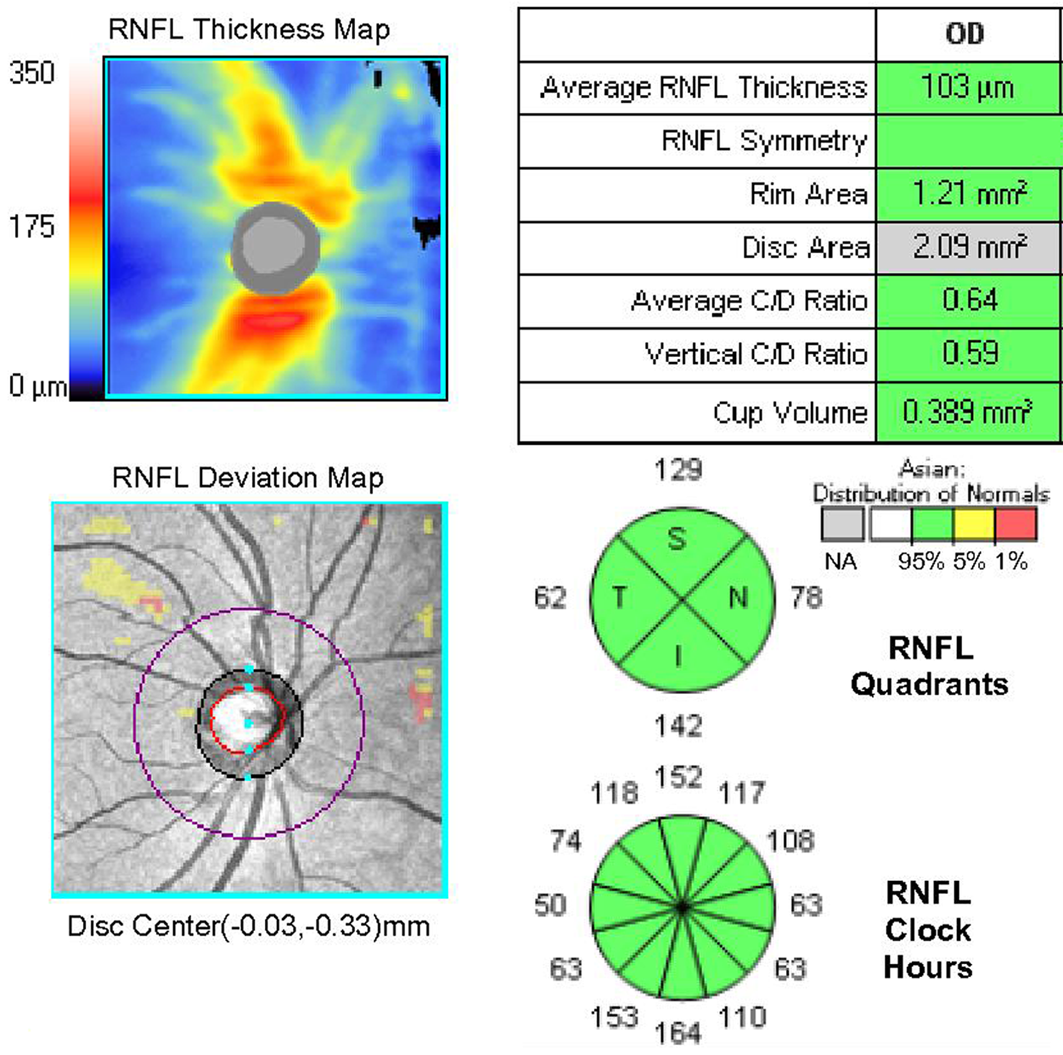

Supplement: S2 Fig — RNFL thickness map (top left) obtains optic disc images through a 6 × 6 × 2 mm3 cube of data using 200 × 200 axial scans. A 3.46-mm diameter circle around the optic disc is shown on the RNFL deviation map (bottom left). The Cirrus HD-OCT system and the algorithm automatically detect the circle and extract a B-scan from it. Significance map (Right) of the same eye shown in S1 Fig. indicates that the RNFL thickness is within the normal limits. (TIF) [file pone.0160448.s002.tif]
